# Exploring the complexities of 1C metabolism: implications in aging and neurodegenerative diseases

**DOI:** 10.3389/fnagi.2023.1322419

**Published:** 2024-01-04

**Authors:** Ayman Bou Ghanem, Yaman Hussayni, Raghid Kadbey, Yara Ratel, Shereen Yehya, Lara Khouzami, Hilda E. Ghadieh, Amjad Kanaan, Sami Azar, Frederic Harb

**Affiliations:** ^1^Faculty of Medicine and Medical Sciences, University of Balamand, Tripoli, Lebanon; ^2^College of Natural and Health Sciences, Zayed University, Dubai, United Arab Emirates; ^3^AUB Diabetes, American University of Beirut Medical Center, Beirut, Lebanon

**Keywords:** one carbon metabolism, folate, Alzheimer’s disease, Parkinson disease, aging, mitochondrial dysfuntion, neurodegenerative disease

## Abstract

The intricate interplay of one-carbon metabolism (OCM) with various cellular processes has garnered substantial attention due to its fundamental implications in several biological processes. OCM serves as a pivotal hub for methyl group donation in vital biochemical reactions, influencing DNA methylation, protein synthesis, and redox balance. In the context of aging, OCM dysregulation can contribute to epigenetic modifications and aberrant redox states, accentuating cellular senescence and age-associated pathologies. Furthermore, OCM’s intricate involvement in cancer progression is evident through its capacity to provide essential one-carbon units crucial for nucleotide synthesis and DNA methylation, thereby fueling uncontrolled cell proliferation and tumor development. In neurodegenerative disorders like Alzheimer’s and Parkinson’s, perturbations in OCM pathways are implicated in the dysregulation of neurotransmitter synthesis and mitochondrial dysfunction, contributing to disease pathophysiology. This review underscores the profound impact of OCM in diverse disease contexts, reinforcing the need for a comprehensive understanding of its molecular complexities to pave the way for targeted therapeutic interventions across inflammation, aging and neurodegenerative disorders.

## 1 Introduction

**Figure 1:**
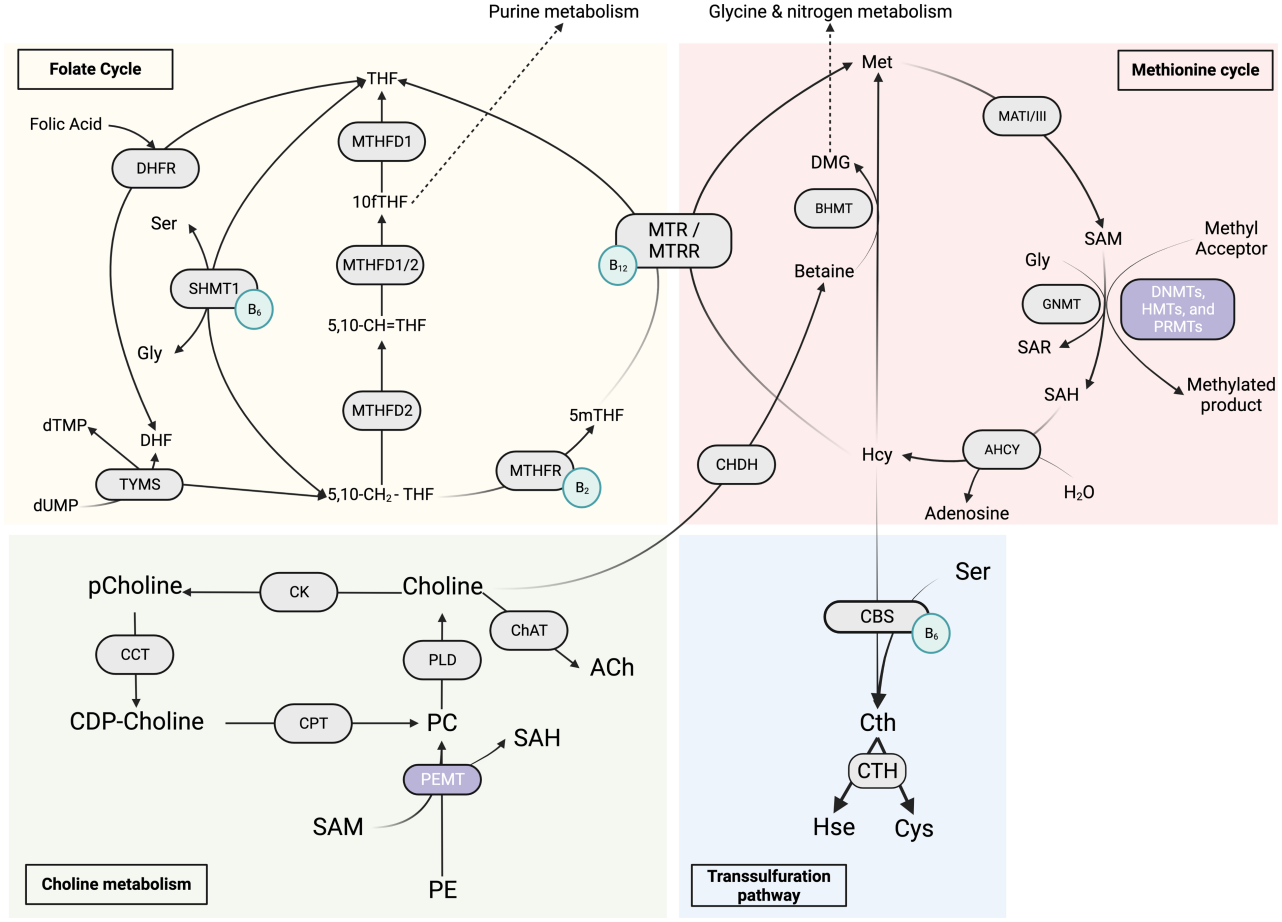
Overview of the “One Carbon Metabolism” metabolic pathways. The red section represents the methionine cycle and its enzymes, the yellow section represents the folate cycle metabolism and its enzymes, the green section represents the choline metabolism and its enzymes, and the blue section represents the transsulfuration pathway. The light green circles represent the coenzymes B_2_, B_6_ and B_12_. 5,10-CH2-THF, 5,10-methylenetetrahydrofolate; 5,10-CH=THF, 5,10-methenyl-tetrahydrofolate; 10fTHF, 10-formyl-tetrahydrofolate; 5mTHF, 5-methyl-tetrahydrofolate; DHF, dihydrofolate; Gly, glycine; Ser, serine; dTMP, thymidine monophosphate; dUMP, deoxyuridine monophosphate; DHFR, dihydrofolate reductase; MTHFD1/2, methylenetetrahydrofolate dehydrogenase; MTHFR, 5,10-methylenetetrahydrofolate reductase; SHMT1, serine hydroxymethyltransferase 1; TYMS, thymidylate synthase; DMG, dimethylglycine; Hcy, homocysteine; Met, methionine; SAH, S-adenosylhomocysteine; SAM, S-adenosylmethionine; Sar, sarcosine; AHCY, S-adenosyl-L-homocysteine hydrolase; BHMT, betaine-homocysteine S-methyltransferase; CHDH, choline dehydrogenase; GNMT, glycine N-methyltransferase; MATI/III, methionine adenosyltransferase; MTR, methionine synthase; MTRR, methionine synthase reductase; Cth, cystathionine; Cys, cysteine; Hse, homoserine; CBS, cystathionine β-synthase; CTH, cystathionine γ-lyase; DNMTs, *de novo* and maintenance DNA methyltransferases; HMT, histone methyltransferase; PRMT, protein arginine methyltransferase; PEMT, phosphatidylethanolamine N-methyltransferase; CDP-choline, cytidine diphosphate-choline; PC, phosphatidylcholine; pCholine, phosphocholine; PE, phosphatidylethanolamine; CCT, CTP:phosphocholine cytidylyltransferase; ChAT, choline acetyltransferase; CK, choline kinase; CPT, cholinephosphotransferase; PLD, phospholipase D.

One-carbon metabolism (OCM) is the process by which one-carbon units in the form of a methyl group is transferred from a donor molecule to various acceptor molecules, thereby assisting in the continuation of many biological reactions that are important for the body homeostasis including cell growth, differentiation, and development. It is a complex network, as represented in Figure 1, of interconnected reactions that includes interlaced folate cycles and activated methionine cycles, both of which are required for cellular activity, emphasizing its importance in health maintenance and tissue repair (Suh et al., 2016). By supplying substrates, it promotes lipid and protein synthesis, oxidation and reduction processes, methylation reactions, and nucleotide anabolism (Locasale, 2013; Yu et al., 2019). Specific amino acids, such as glycine, serine, and threonine, can be used to obtain the methyl groups that make up the core of OCM. The serine synthesis pathway (SSP) allows for the delivery of these amino acids into the cells as well as their *de novo* production using glucose (Locasale, 2013; Tsun and Possemato, 2015; Yu et al., 2019). These methyl groups take the form of S-adenosylmethionine (SAM), which acts as the universal methyl donor in methylation reactions. *In vivo*, SAM is synthesized by l-methionine adenosyltransferase (MATs) from adenosine triphosphate (ATP) and l-methionine (l-Met) during the methionine cycle (Popadić et al., 2021). S-adenosylhomocysteine (SAH) is formed after the methyl group is transferred from SAM to an acceptor substrate (Mentch and Locasale, 2016). OCM can create cofactors required in many biochemical and metabolic pathways, including redox electron carriers such as NADH and NADPH. In addition, it can produce ATP (Vazquez et al., 2011). Thus, it should come as no surprise that deviating from OCM increases our risk of aging and age-related disorders (Lionaki et al., 2022). Furthermore, the production of serine/glycine and one-carbon metabolism are critical in the survival and rapid multiplication of carcinogenic cells, hence their significant potential therapeutic implication. Excessive stimulation of serine/glycine synthesis promotes cancer by supplying a single-carbon unit for one-carbon metabolism (Pan et al., 2021). As a result, different dietary inputs act as precursors for one carbon unit, which subsequently serves as a building block for biosynthesis, methylation, and redox activities (Rosenzweig et al., 2018). Nutritional and/or genetic imbalance of the OCM can affect the developmental process, resulting in decreased embryo growth and poor pregnancy outcomes (Clare et al., 2019).

Unlike prokaryotic one-carbon metabolism (OCM) activities, eukaryotic OCM processes are compartmentalized, with each compartment using a different metabolic route. Regardless of the route, folate cofactors always mediate these pathways. In contrast to most bacteria, yeast, and plants, mammals-including humans- cannot synthesize folate and therefore rely on dietary folate consumption to maintain stable levels (Ducker and Rabinowitz, 2017). The synthetic food additive folic acid (vitamin B9) is usually the major source of folates for humans (Mason, 2003). A shortage of folate intake causes adult anemia and predisposes newborns to a range of neural tube defects (Ducker and Rabinowitz, 2017).

Folic acid must first be metabolized to an intermediate dihydrofolate (DHF) and then via the enzyme dihydrofolate reductase (DHFR) to tetrahydrofolate (THF). THF is the physiologically active form of folates that serves as a methyl donor before it can access one of the carbon metabolism routes. THF carries chemically reactive one-carbon units, primarily amines (serine and glycine) and choline breakdown products such as dimethylglycine and methylglycine (Anderson et al., 2011; Ueland, 2011). In most human tissues, the reduced form of folate 5-methyl tetrahydrofolate is the primary form of one carbon-loaded folate (Clare et al., 2019). 5-methyl tetrahydrofolate can be covalently modified by enzymes found in the cytosol and mitochondria. They modify the covalent link through which the one-carbon unit is attached in order to interchangeably convert between various oxidation states. Carbon may undergo three distinct oxidation states, and each of these oxidation states eventually donates one carbon unit to power separate biochemical processes (Tibbetts and Appling, 2010).

The role of mitochondria in the oxidation of one-carbon donors came to light in the early 1950s. Several studies revealed the role of folate-dependent enzymes in mitochondrial activities. Subsequently, the discovery of these enzymes established a connection between cytoplasmic and mitochondrial one-carbon metabolism. At any given time, the mitochondria contain around 40% of total cellular folate (Shin et al., 1976). The difficulty of one-carbon loaded folates to cross intracellular membranes has been recognized as a distinguishing property of one carbon metabolism. Therefore, it is crucial to be able to create these molecules both inside the mitochondria and in the cytoplasm (Anderson et al., 2011). Serine, glycine, and formate have been identified as molecules that link the reactions occurring in the two compartments (Cybulski and Fisher, 1977; Palmieri, 2004). Most of the one-carbon flow within mitochondria involves an oxidative process, leading to the production of formate from the single-carbon units obtained from these connector molecules (Lewis et al., 1978). Hence, it is believed that the mitochondrial system serves as the main channel for creating glycine and single-carbon units, which are essential for cell division (Barlowe and Appling, 1988; Desler et al., 2010).

Cellular division and a variety of other biological processes require B vitamins operating as cofactors, precursors, and substrates. B vitamins are made up of eight water-soluble vitamins: B1, B2, B3, B5, B6, B7, B9, and B12. These B vitamins are grouped based on their capacity to dissolve in water and their interdependent roles in cellular coenzyme functions, rather than chemical structure similarities. Consuming proper amounts of B vitamins in our diet is critical for human health preservation (Lyon et al., 2020). Deficiencies in these vitamins can have serious health repercussions if there is a disturbance in the pathway (Porter et al., 2016). Most B vitamins are involved in one-carbon metabolism, either directly or indirectly (Lyon et al., 2020).

This review article will first provide an overview of the one carbon metabolism in several biological processes such as inflammation and aging, then focus on the implication of OCM in neurodegenerative diseases and more particularly Alzheimer’s and Parkinson diseases.

## 2 1C metabolism implication in physiological processes (aging and inflammation)

Because OCM lies at the heart of many cellular metabolisms, metabolic dysregulations have been associated with several age-related diseases such as cardiovascular disease (CVD), cancer, inflammation, and neurocognitive disorders. Notably, changes in OCM are significantly associated with the aging process.

### 2.1 OCM and aging

Aging is the deterioration of several physiological functions that are crucial for survival and reproduction. Gilbert cited several causes leading to aging: normal metabolism (aging being a byproduct), accumulated reactive oxygen species (ROS), wear-and-tear, and genetic instability (Gilbert, 2000). In a publication by Harman, he explored the hypothesis suggesting that free radicals might have a notable impact on the aging process. This influence is proposed to occur through the impairment of crucial cellular components such as DNA, proteins, and lipids. Over time, this damage may contribute to cellular dysfunction, ultimately leading to the aging phenomenon (Harman, 1956). The term “aging” describes the gradual loss of various physiological processes essential for survival and reproduction. Furthermore, the importance of folate (vitamin B9) metabolism in the aging process of animals has been underlined by a number of observations (Lionaki et al., 2022). The level of folate in a person’s blood decreases with age, hastening the aging process. As a result, there is generally a 50% decrease in the blood folate levels in aging people. In studies using mice provided with a diet low in folate, aging-related symptoms such as short-term memory loss and altered S-adenosylmethionine (SAM) metabolism were observed. However, providing the mice more folate had a positive impact on their longevity (Lionaki et al., 2022). The essential amino acid methionine, on the other hand, has revealed an intriguing inverse connection with aging. According to Kitada et al. extending lifespan has been accomplished by reducing methionine concentration or limiting its consumption within a diet (more precisely, a calorie-restrictive diet, as opposed to malnutrition; Kitada et al., 2021). Growth hormone (GH) and ROS concentrations were lowered by methionine restriction (MR), which enhanced insulin sensitivity and decreased mitochondrial DNA damage, respectively (Lionaki et al., 2022). Overall, these findings reveal possible routes for therapies to support healthy aging and shed insight on the intricate interactions between folate metabolism, methionine restriction, and the aging process.

### 2.2 OCM and inflammation

Inflammation is a biological response of the immune system triggered by multiple factors such as pathogens (bacteria, viruses, parasites, fungi), damaged cells, and toxic compounds. Inflammation is a defense mechanism that maintains health and homeostasis by removing the trigger and mediating healing (Chen et al., 2017). Macrophages are innate immune cells that respond to pathogens and take part in tissue repair. They contribute a major role in inflammation by adapting their metabolic pathways to drive a pro-inflammatory phenotype (Watanabe et al., 2019). Once the new phenotype is acquired, both methionine and folate cycles will be uncoupled since methionine plays a far more important role in inflammation than the folate cycle (Yu et al., 2019). This will result in generating SAM from methionine within the methionine cycle. The literature is rife with evidence that correlates methionine and SAM to Lipopolysaccharide (LPS)-induced inflammation. Yu et al. consider SAM as a key metabolite stimulating inflammation induced by LPS, by activating the pro-inflammatory genes such as interleukin 1 beta (IL1-β; Yu et al., 2019). Any impairment of the metabolic pathway generating SAM will result in an anti-inflammatory outcome. However, Coleman et al. consider an improvement in immune cell function with methionine supply (Coleman et al., 2021). Scientists have found that administration of Rumen-protected methionine (RPM) and SAM reduces inflammation and inflammatory mediators caused by LPS, increases neutrophil capacity to phagocytose, and is associated with lower pro-inflammatory genes IL1-β expression (Ji et al., 2019). Inflammation induced by disturbances in one-carbon metabolism can set in motion a series of events that contribute to the pathogenesis of Alzheimer’s and Parkinson’s diseases, including altered gene expression, chronic inflammation, and oxidative stress, ultimately influencing the onset and progression of these neurodegenerative disorders. Thus, OCM seems to play an important role in reducing inflammation.

## 3 1C metabolism and Alzheimer’s disease

**Figure 2:**
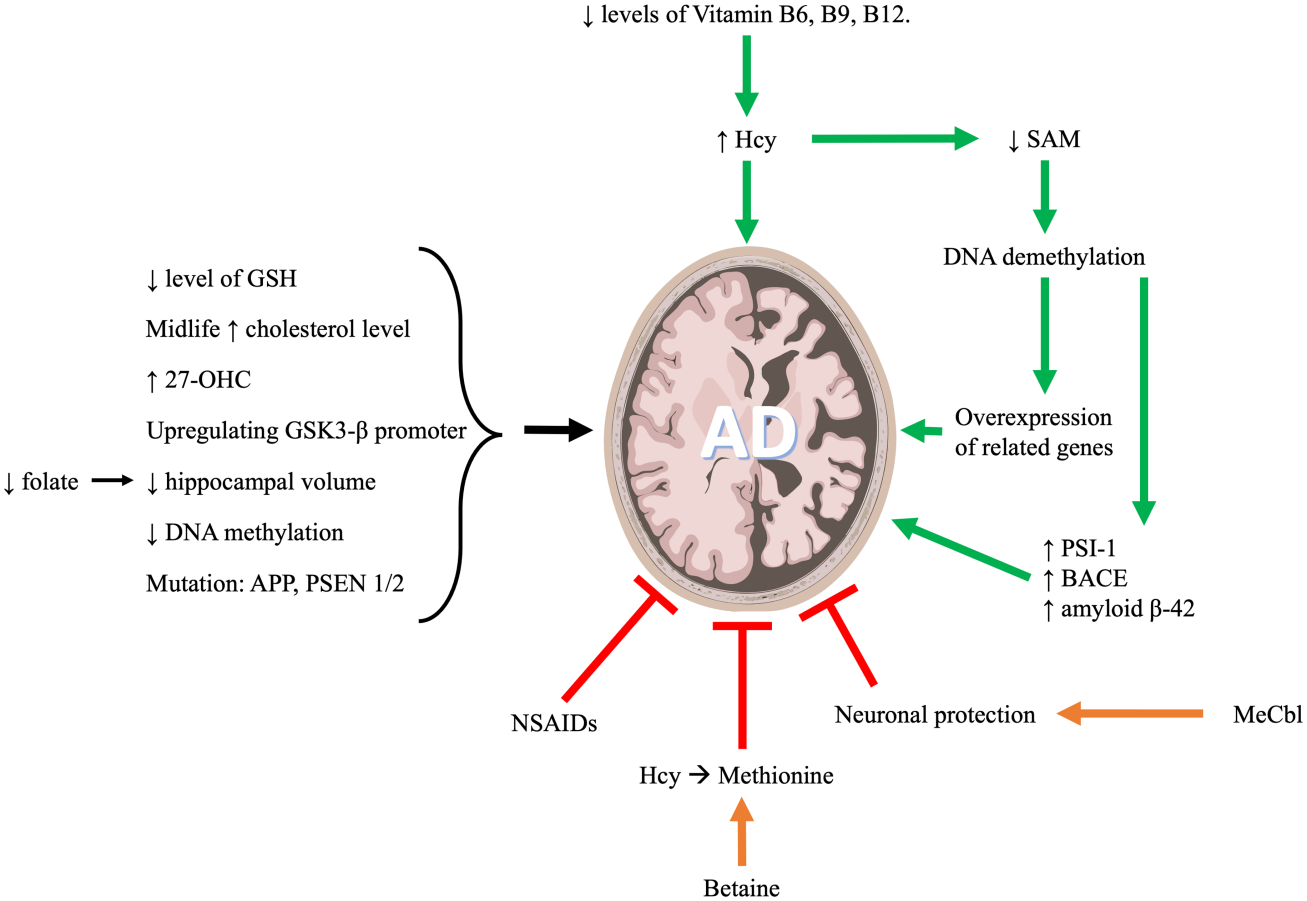
One carbon metabolism and its implication in Alzheimer’s disease progression.

AD is a form of neurodegenerative dementia associated with cognitive and functional decline that eventually results in death. The primary pathologies of this disease include neuronal beta amyloid plaques and hyperphosphorylated tau protein in neurofibrillary tangles (NFTs; Soria Lopez et al., 2019). OCM metabolites are highly implicated in AD as indicated in Figure 2. In what follows we will discuss the role of OCM pathway in the development and progression of AD.

### 3.1 B vitamins (B12, B6 or others if found) in late onset AD

There is a documented relationship between Hcy B vitamins and an increased risk of cognitive impairment and AD. Hcy is an amino acid that is naturally produced in the body and its metabolism requires folate (vitamin B9), pyridoxine (vitamin B6) and cobalamin (vitamin B12). These vitamins are involved in converting Hcy into other metabolites that will be used systemically. Deficiencies in these vitamins are associated with elevated levels of Hcy which in turn is linked with increased risk of AD (Bhargava et al., 2018).

Hcy can be converted to cystathionine by the enzyme cystathionine beta-synthase (CBS) which requires pyridoxine (B6) as a cofactor. Cystathionine is then converted to cysteine by the enzyme cystathionine gamma-lyase (CGL) that requires vitamin B6 as well. Deficiency in B6 can disrupt the function of these two enzymes and thus, cause elevated levels of Hcy. Another pathway for Hcy to be metabolized is the conversion of Hcy to methionine and in this pathway both folate (B9) and cobalamin (B12) are required (Hankey, 2018). Thus, a deficiency in both B9 and B12 may elevate Hcy levels and increase the risk of AD.

The cognitive decline in AD can be predicted by plasma levels of one-carbon metabolites (Dayon et al., 2017). Plasma levels of folate, vitamin B12 and Hcy are strongly associated with cognitive function in cognitively impaired elderly suffering from AD (Kim et al., 2014). Nutritional deficits in B vitamins will eventually lead to hyperhomocysteinemia, consequently, this will decrease SAM level. SAM is a methyl donor. Therefore, a reduced level of SAM can induce demethylation of DNA resulting in overexpression of genes that are involved in the AD pathology (Marques and Outeiro, 2013). Amyloid precursor protein (APP) processing and beta-amyloid (A beta) production involve DNA methylation through the regulation of Presenilin 1 (PS1) expression (Fuso et al., 2005). Exogenous S-adenosylmethionine (SAM) can reduce *in-vitro* PS1 expression, synthesis, and amyloid beta 1–40 peptides production. These effects are likely due to the methylation of a CCGG site in PS1 promoter (Scarpa et al., 2003). Similarly, Fuso et al. confirmed that PS1 and BACE (beta-secretase) are both regulated by methylation (Fuso et al., 2005). Thus, a reduction in vitamin B12 and folate in the culture medium can lead to a reduced level of SAM which consequently increases PS1 and BACE levels, and eventually increasing amyloid beta production (Fuso et al., 2005). Moreover, it was shown that patients with Late-Onset Alzheimer’s Disease (LOAD) have decreased levels of SAM in the cerebrospinal fluid (CSF), which affects the apolipoprotein E4 carriers (Linnebank et al., 2010). Apolipoprotein (Apo) E is a protein central to lipid metabolism, neurobiology and neurodegenerative diseases. The major function of ApoE is mediating the binding of lipoproteins in the plasma or interstitial fluids to specific cell-surface receptors. There are 3 major isoforms of this protein which are apoE2, apoE3, and apoE4. The 3 APOE alleles have protein isoforms that differ only at 2 amino acid positions (112 and 158). The ε4 allele of ApoE gene (ApoE4) contributes to the risk factor for AD (Martens et al., 2022). Moreover, In a 2018 investigation by Prendecki et al., it was revealed that specific genetic variations, such as rs1052452 and rs2075650 in the TOMM40 gene, and rs4420638 in the APOC1 gene, were linked to an elevated susceptibility to Alzheimer’s disease (AD). This heightened risk coincided with increased levels of homocysteine (Hcy) and reduced levels of glutathione (GSH), resulting in observable oxidative DNA damage indicated by elevated levels of 8-oxo-2′-deoxyguanosine (8-oxo2dG) (Prendecki et al., 2018). Additionally, a subsequent study by Prendecki et al. (2019) explored the association between biochemical parameters and APOE genetic variants in AD patients. The findings indicated that the levels of apoE, miR-107, miR-650, and Hcy exhibited variations in Polish AD patients compared to the control group. ApoE, miR-107, and miR-650 demonstrated decreases in AD patients, while Hcy exhibited an increase. This pattern could be attributed to the interplay between APOE E4 and Hcy, leading to heightened oxidative stress and DNA damage. Furthermore, the study proposed that miR-650 might be a promising target for future research in AD patients (Prendecki et al., 2019).

Hyperhomocysteinemia (HHcy) is one of the modifiable risk factors that are associated with AD (Quadri et al., 2004; Smith, 2008; Van Dam and Van Gool, 2009). Hcy can cause damage and death in the neurons, thus, impairing short-term memory and learning (Škovierová et al., 2016). A meta-analysis study reported that AD patients have higher levels of Hcy and lower levels of folate as compared to patients with no dementia. Quantitatively, it has been reported that for every 5 μmol/L increase in the level of plasma Hcy, there is a 12% increase in the risk of AD (Wang et al., 2021). Elevated homocysteine is associated with white matter damage, brain atrophy, cognitive decline, neurofibrillary tangles, and dementia (Smith and Refsum, 2016). These findings are echoed through the literature, where another study evaluating the effects of baseline iron, cysteine, and Hcy on brain atrophy rate, showed that Hcy was associated with accelerated brain atrophy and predicted worse cognition. The same study demonstrated that the brain atrophy rate was abrogated by treatment with B vitamins (Jakubowski et al., 2021). Regardless of the severity of the dementia in AD, there is approximately a one-third increase in the level of Hcy in the blood of these patients. This increase is robustly associated with a decrease in the level of blood folate, but not that of vitamin B12 (Zhang et al., 2022). The brain nutrient status appears to be mirroring the circulatory nutrient status. Therefore, AD patients display lower CSF levels of folate and vitamin B12, which is likely linked with the poor recognition and neurological problems (Mohajeri et al., 2015). Although, isolated vitamin B12 deficiency may not be associated with AD, the combination of low folate and vitamin B12, in addition to elevated levels of Hcy, is linked to AD in older Chinese patients (Ma et al., 2017). Various other combinations of folate, vitamin B12 and Hcy levels have been correlated with AD. for example, low levels of vitamin B12, with normal Hcy level and normal folate were shown to be associated with AD. Moreover, the combination of high Hcy, low folate, and normal vitamin B12 was also linked to AD. Finally, another study revealed that high Hcy level, low vitamin B12 and any folate level was associated with AD as well (Chen et al., 2015). Thus, it is evident that the exact role that each nutrient component, circulating and/or central, plays in the development and progression of AD remains to be elucidated (de Wilde et al., 2017).

Nevertheless, low folate levels are also associated with shorter telomeres, most likely a result of DNA damage in the telomeric region. Telomere length is epigenetically regulated by DNA methylation and is directly influenced by the status of folate in the body (Panossian et al., 2003). Shortening of telomeres cap is often an indicator of aging and possibly linked to load and cognitive decline. The improvement in cognitive function, in patients with AD, using folic acid, B6 or B12 supplementation may be due to this telomeric effect (Chen et al., 2016) and/or the reduction plasma levels of Hcy (Malouf et al., 2003; Sun et al., 2007; Gargari et al., 2011).

There is no dearth of studies linking folate, B vitamins, Hcy and Alzheimer’s. Higher dietary intake of B vitamins lowers serum levels of Hcy, the risk of AD and cognitive dysfunction (Dangour et al., 2010; Cheng et al., 2016; Pi et al., 2020). Quantitatively, with elevated folate levels, the risk of developing AD decreased by 9% (Moretti et al., 2017). Moreover, supplementation with folate, cobalamin and pyridoxine in elderly patients facing cognitive decline demonstrated a reduction in gray matter regions that are vulnerable to AD (McCully, 2016). It should be noted though, that inappropriate administration of folate when there is a vitamin B12 deficiency may cause neurologic and hematologic relapse (Reynolds, 2014). In conclusion, folate deficiency is a risk for AD while folate intake is a protective measure against AD that needs to be considered (Zhang et al., 2021).

Supplementation with methylcobalamin (MeCbl), the activated form of vitamin B12, has been used as an auxiliary treatment for AD. It promotes neuronal regeneration of nerves and antagonizes glutamate neurotoxicity (Zhang et al., 2013). Other experimental therapeutic cocktails including 5-methyltetrahydrofolate, methyl B12, betaine, and N-acetylcysteine (NAC) may help target multiple pathways simultaneously in order to decrease the total serum Hcy and its toxicity (Leon et al., 2018).

In summary, a deficiency in B vitamins and the ensuing elevated Hcy levels and SAM depletion may contribute to late-onset of AD. This is achieved through a complex interplay of multiple mechanisms that involve Hcy metabolism, oxidative stress, neurodegeneration, and SAM-dependent epigenetic modifications.

### 3.2 Neurotoxicity

OCM is a foundational pillar of normal cell growth and function. Neuronal tissue, especially of the mammalian type, is well known for its high metabolic demands. Therefore, deficient OCM pathways lead to considerable neuronal damage and toxicity (Table 1).

**Table 1:** Role of various elements within the 1C metabolism in Alzheimer’s disease.

Component	Description	Effect	References
Neurotoxicity	Methyl donor deficiencies Excess homocysteine Decrease levels of β-synthase Increase levels of 5-methylcytosine Missense mutation in MTHFR Folate deficiency	Depressed neural activity Neuronal immaturity Decreased dendritic complexity Reduced dendritic maturation in Dentate Gyrus cells decreased Decreased hippocampal neurogenesis Promotion of pro-inflammatory microglial profile Cell cycle arrest at G0/G1 phase ➔ pro-apoptotic profile DNA methylation ➔ upregulation of Sprouty-2 ➔ impairment of fibroblast growth	Rabaneda et al. (2016), Yang et al. (2016), Liu et al. (2021), Pi et al. (2021), Alachkar et al. (2022), Araki et al. (2022), and Kalecký et al. (2022)
Neurotransmitter	Disruption of choline metabolism by homocysteine Shift of GluN2A-NMDAR by homocysteine Activation of the matrix metalloproteinases (MMPs) Folate deficiency Decrease of H2S production	Decrease levels of acetylcholine ➔ cognitive decline Pro-inflammatory profile in neurons Increase of Ca^2+^ influx ➔ cell death Antagonization of GABA-A receptor ➔oxidative damage Impairment of S-adenosylhomocysteine hydrolysis ➔ decrease in adenosine production Endoplasmic reticulum stress- mediated toxicity Tau hyperphosphorylation	Selley (2004), Lominadze et al. (2012), Li et al. (2014), Bahous et al. (2019), Rajagopal et al. (2019), Babaei (2021), and Giovinazzo et al. (2021)
Glial cells	Homocysteine levels	Compromised glutamate uptake Disruption in the redox/mitochondrial homeostasis Altered inflammatory response Impaired cell signaling Gliotoxicity Compromise of astrocyte function Cytoskeletal changes of glial cells (filopodia) ROS production in astrocytes Cell death	Maler et al. (2003), Loureiro et al. (2010a,b) Longoni et al. (2018), Pierozan et al. (2018), and Wyse et al. (2021)
Oxidation	Homocysteine levels Decrease of H2S production Folate deficiency	Oxidative stress ➔ pro-apoptotic state Mitochondrial damage DNA damage	Kruman et al. (2000), White et al. (2001), Tchantchou et al. (2006), Wang et al. (2016), Kumar et al. (2018), and Koohpeyma et al. (2019, 2020)

One of the major markers of disrupted OCM in AD is elevated Hcy (Wang et al., 2021; Zuin et al., 2021). Hcy is a known neurotoxic compound (Obeid and Herrmann, 2006). Hippocampal neurons cultivated in high Hcy or low folate media consistently showed decreased cell survival (Kruman et al., 2005; Araki et al., 2022), as well as reduced maturity and connectivity (Araki et al., 2022). Other neural populations were also involved in folate deficiency (FD)-mediated neurotoxicity. These included cerebellar Purkinje cells that suffered increased apoptosis and decreased neurite development (Oldreive and Doherty, 2007). Purkinje cells are of known significance in the etiology of the cognitive and behavioral sequelae of Alzheimer’s (Mavroudis et al., 2019). Moreover, FD’s effect on less differentiated neuronal lines (Kruman et al., 2005; Wang et al., 2016) is of special importance since neurogenesis, especially at the level of the hippocampus, is fundamental to cognitive performance and dementia (Chen et al., 2023; Salta et al., 2023). At a systemic level, folate deficiency led to decreased hippocampal volume in Alzheimer’s patients even when confounding factors such as age and severity of amyloidosis were accounted for (Choe et al., 2014; Yang et al., 2016). The findings of Firbank et al. corroborate these claims but implied that folate deficiency was not the proximal cause of this atrophy, instead pointing towards the HHcy resulting from this lack of folate (Firbank et al., 2010).

Despite preliminary evidence pointing towards their significance (Dayon et al., 2017), other elements of the OCM pathway, namely those of the methionine cycle, have traditionally received relatively limited academic attention. A fact that is changing as the depths of OCM yield themselves to modern forays. The third figurative arm of OCM: The trans-sulphuration pathway, despite its relevance in neurotoxicity, its effects mostly act within the paradigm of oxidative stress.

Within the methionine cycle, Hcy is normally converted into methionine to regulate the latter’s levels (Ducker and Rabinowitz, 2017). Recently conducted research showed that high methionine led to decreased hippocampal neurogenesis and promoted a pro-inflammatory microglial profile (Alachkar et al., 2022). Pi et al. delved into the mechanistic facets of methionine and proposed that elevated 5-methylcytosine, a transcription factor, could be a possible mediator of methionine-mediated neuropathy (Pi et al., 2021). Aside from the effects on general neuronal survival, it was found that high methionine levels led to decreased cell survival, increased amyloid-β (Aβ) and amyloid-β-associated proteins expression and decreased the expression of enzymes involved in Aβ metabolism. Another effect of elevated methionine is the increase in Hcy, probably due to a shift in equilibrium. Regardless, excess methionine levels can thus have downstream Alzheimer’s-associated consequences that extend far beyond the effect of methionine itself.

Betaine is a methylated derivative of glycine and is a significant methyl donor in OCM (Ducker and Rabinowitz, 2017). Betaine is involved in the conversion of Hcy into methionine. In this capacity, it serves to clear excess Hcy and thus conveys a neuroprotective effect (Chai et al., 2013). This was further demonstrated in the work of Sun et al. where betaine ameliorated pro-inflammatory signaling mediated by malnutrition-induced high Hcyin AD patients (Sun et al., 2017). Mechanistically, betaine had measurable and complex effects on Aβ accumulation and Tau phosphorylation, key chain-links in AD pathology. Betaine prevented Aβ plaque formation *in vitro* (Leon et al., 2018) as well as recovered motor function in *C. elegans* models of Aβ amyloidosis (Leiteritz et al., 2018). In murine models, Aβ-injected mice maintained their cognitive function when treated with betaine (Ibi et al., 2021) in a process that involved Sirtuin 1 (Ibi et al., 2022).

The adoption of a more holistic understanding of OCM, alongside recent improvements in modeling and analysis (Guiraud et al., 2017), will hopefully herald future works that will build a more coherent understanding of this pathway’s ubiquitous role in neurodegenerative pathologies leading to possible novel therapeutic routes.

### 3.3 Oxidation

The role of oxidation in AD is well established in academic literature (Tönnies and Trushina, 2017; Ionescu-Tucker and Cotman, 2021; Bai et al., 2022). Neurons are especially sensitive to oxidative stress, the burden of oxidation usually leading to disrupted cell function and even death (Salim, 2017). The neurotoxic dimensions of broad disruptions in 1-C metabolism have already been elucidated in depth in the Neurotoxicity section. This section will limit itself to the association between AD and transsulfuration pathway.

OCM can be dissected into three conceptual components: the folate and methionine cycles in addition to the so-called trans-sulfuration pathway (TSP). The latter pathway is of special significance in the context of oxidation and oxidative stress in AD. Briefly, the TSP includes the condensation of Hcy and serine into cystathionine under the action of cystathionine-β-synthase (CβS). Cystathionine is then hydrolyzed by cystathionine γ-lyase (CGL) into cysteine in a reaction that yields 𝛼-ketobutyrate and ammonia (Yudkoff, 2012). As will be discussed below, the clearing of Hcy as well as the synthesis of various TSP products are important contributors to the various anti-oxidative pathways within the framework of AD and other neurodegenerative diseases (Kamat et al., 2016).

Cystathionine-β-synthase is the first step in the induction of Hcyinto the TSP. Indeed, animal CβS knockouts are a popular model of HHcy (Troen, 2005; Pacheco-Quinto et al., 2006; Zaric et al., 2019), and a veritable body of literature has demonstrated the strong association between impaired CβS function and AD-associated cognitive decline (Leiteritz et al., 2018; Elsherbiny et al., 2020). There is some evidence that similar mutations may play a role in the etiology of AD in humans (Beyer et al., 2004; Obeid and Herrmann, 2006). Kalecký et al. showed that Parkinson’s (PD) patients on levodopa^®^, a known depleter of B vitamins, had a higher incidence of AD-like cognitive decline. At a molecular level, CβS activation was found to be impaired only in demented PD patients (Kalecký et al., 2022).

Aside from its direct role as a ROS scavenger, cysteine from the trans-sulfuration pathway is essential in the synthesis of the most important antioxidant in the human body: glutathione (GSH; McBean, 2012). In this tripeptide molecule, the cysteine residue is central to both the enzymatic and non-enzymatic roles due to its thiol group’s ability to undergo reversible redox reactions. Impairments in the production and maintenance of adequate GSH levels have long been known to be associated with Alzheimer’s severity (Haddad et al., 2021; Chen et al., 2022). As such, OCM exerts an antioxidative effect by promoting cysteine, and subsequently glutathione production (Mosharov et al., 2000; Vitvitsky et al., 2006), leading to prevention and possible amelioration of AD-associated cognitive decline (Haddad et al., 2021).

Cysteine derived from the trans-sulphuration pathway can also contribute to the synthesis of hydrogen sulfide (H_2_S). H_2_S is traditionally known as a toxin due to its ability to block complex IV of cellular respiration. However, its role in cellular signaling, especially in the context of neurodegenerative diseases, has emerged recently (He et al., 2019). While H_2_S does in fact have a direct ROS scavenging role, the more significant aspect of its antioxidant effect is mediated by its ability to up-regulate the production of endogenous antioxidants including superoxide dismutase (SOD), catalase, and glutathione peroxidase (GPx) as well as the aforementioned GSH (Tabassum et al., 2020). H_2_S also promotes a shift towards an anti-inflammatory profile which decreases inflammation-associated oxidative stress (Salmina et al., 2015). Additionally, H_2_S alleviates so-called endoplasmic reticular (ER) stress (Zhong et al., 2020). ER stress refers to the saturation of the protein-folding process due to the accumulation of misfolded proteins in various diseases including AD. As such, H_2_S is theorized to be essential in mitigating this central pathogenic stream. Additionally, H_2_S’s inhibitory effect on cellular respiration may play a role in diminishing respiration-associated ROS release. The literature vastly focuses on H_2_S as a major mediator in the antioxidative effect of the TSP. However, a cohesive understanding of the entire pathway and its complex role in the oxidative dimensions of Alzheimer’s remains just beyond reach, presenting the potential inquirer with a fertile ground for further work.

### 3.4 Alteration of cholesterol and lipids

OCM, encompassing the folate cycle, methionine cycle, and trans-sulfuration pathway, plays a crucial role in providing one-carbon (1C) units used for the biosynthesis of nucleic acids, proteins, and lipids necessary for growth. Even though there is no clear relationship between OCM and cholesterol, it is important to develop the implication of altered levels of cholesterol in neurodegenerative diseases like AD. When investigating the relation between cholesterol and AD, it was shown that serum total cholesterol, in midlife, is associated with an increased risk of AD (Solomon et al., 2007). In contrast to previous studies, high cholesterol in late life was linked with a decreased risk for dementia (Mielke et al., 2005). These conflicting results might be explained by cholesterol measurement timing, age, and the clinical onset of dementia (Mielke et al., 2005). Another study generated similar results indicating that high midlife serum total cholesterol is a risk factor for subsequent AD, but decreasing serum total cholesterol level after midlife may indicate an ongoing disease process and thus may indicate a marker of late-life cognitive impairment (Solomon et al., 2007). However, a recent analysis, based on thirty-four articles linking total serum cholesterol with AD, indicated that there is evidence that suggests that midlife total serum cholesterol increases risk of late-life AD (Anstey et al., 2017).

Furthermore, a meta-analysis was performed to assess the importance of serum levels of total cholesterol (TC), low-density lipoprotein cholesterol (LDL-C), triglycerides (TG) and high-density lipoprotein cholesterol (HDL-C) in AD. The results showed an increased level of serum TC and LDL-C and no significant changes in HDL-C and TG levels. This suggests that elevated TC and LDL-C levels might be a potential risk for cognitive impairment (Liu et al., 2020).

Cholesterol in the central nervous system (CNS) comes, almost entirely, from endogenous synthesis as the circulating cholesterol is unable to cross the blood–brain barrier (BBB). Astrocytes are more active than neurons in synthesizing cholesterol and this renders the neurons dependent on cholesterol delivery from nearby cells for neurite extension, axonal regeneration, and synaptogenesis. Cholesterol is mainly transported by HDL-like lipoproteins associated with apoE within the brain. However, although CNS cholesterol content does not depend on dietary or hepatic synthesis, there is a relationship between neurodegenerative disorders like AD and plasma cholesterol levels (Giudetti et al., 2016).

24S- and 27-hydroxycholesterol (24S and 27-OHC) are side-chain oxidized cholesterol metabolites, oxysterols, that can cross the BBB. 24S-OHC is produced by 24-hydroxylase and is a result of the endogenous brain synthesis, whereas 27-OHC comes from the circulation, crosses the BBB and is then transformed into 7α-hydroxy-3-oxo-4-cholestenoic acid (7-OH-4-C) by the enzyme CYP7B. Then, 7-OH-4-C passes through the BBB and goes to the liver to be eliminated (Björkhem et al., 2009; Wood et al., 2014). The production of 24S-OHC is a way for the brain to tightly control the brain cholesterol levels (Giudetti et al., 2016). Thus, the major source of the circulating 24S-OHC is the brain and therefore, plasma levels of 24S-OHC reflects the number of metabolically active neurons which in turn reflects the volume of the gray matter structures. Plasma 24S-OHC was found to be reduced proportionally to the degree of brain atrophy in AD as measured by MRI (Leoni and Caccia, 2011).

As stated previously, neurons rely mainly on the delivery of cholesterol from nearby cells. Neurons down-regulate their cholesterol synthesis and receive cholesterol from ApoE lipoproteins secreted by astrocytes. ApoE transcription is regulated by 24S-OHC whereas, 27-OHC is formed outside the brain and crosses the BBB proportionally to the barrier dysfunction (Leoni and Caccia, 2011). In addition, other studies have shown that in the brains of patients with AD, there is an accumulation of 27-OHC and a reduction in 24S-OHC (Leoni et al., 2002; Shafaati et al., 2011). Those changes were said to be modified by the cholesterol levels in the circulation (Björkhem et al., 2006; Shafaati et al., 2011). Moreover, many other oxysterols can participate in cholesterol metabolism and the levels of these were increased in the brains of late AD patients, including 25-hydroxycholesterol (25-OHC), 7-ketocholesterol (7-K), 7β-hydroxycholesterol (7β-OH), 5α,6α-epoxycholesterol (α-epoxy), 5β,6β-epoxycholesterol (β-epoxy), 4α-hydroxycholesterol (4α-OH), and 4β-hydroxycholesterol (4β-OH; Testa et al., 2016). 25-OHC is the most cytotoxic agent among the cholesterol oxides in microglial cells. It has been demonstrated as a risk factor for AD as it assists in the intracellular transport of Aβ peptides (Sharma et al., 2017). Furthermore, less than 1% of 24S-OHC is excreted in the cerebrospinal fluid (CSF). This fraction is an indicator of neuronal damage and rate of neuronal loss. In CSF of patients with AD, there is an increased level of 24S-OHC. This concentration in the CSF is correlated with CSF ApoE, cholesterol, and tau. During the process of neurodegeneration, it is likely that excess cholesterol was converted into 24S-OHC in the neurons, leading to the up-regulation and increased expression of ApoE proportionally to the amount of neurodegeneration (Leoni and Caccia, 2011).

Cholesterol, along with sphingolipids, are integral components of lipids rafts (LR). LRs are microdomains in the membrane that create cell-signaling platforms essential for neuronal functions. Lipid rafts have three vital roles in AD which are promoting the generation of Aβ peptide, facilitating its aggregation on membranes of the neurons to form toxic oligomers, and having certain neuronal receptors that are responsible for the transduction of AD-related neurotoxicity and memory impairment of the Aβ oligomers (Rushworth and Hooper, 2011). These cholesterol enriched LR are the main reason behind Aβ aggregation (Rushworth and Hooper, 2011), indicating that a high central or peripheral level of cholesterol would disturb the process of Aβ fibrillation (Ariga et al., 2011). A key step in the onset of AD is the conversion of the soluble, nontoxic Aβ protein into an aggregated, toxic form that is rich in β-sheets. It is suggested that Aβ induces changes in neuronal membrane fluidity due to its interactions with components in the membrane such as gangliosides, cholesterol, and phospholipids. Gangliosides (GM), especially GM1, binds to Aβ and converts it into the toxic, aggregated form (Ariga et al., 2011). Moreover, if the cholesterol content in the neuronal membrane increases, then this will accelerate the binding of Aβ to GM1 and thus, promote Aβ fibrillation. Cholesterol will form a hydrogen-bond between its OH group and the glycosidic-bond that is linking the glycine part of GM1 to ceramide, thereby creating a tilt in the head group of this glycolipid. This change stabilizes the active conformation of the GM1 dimer whose head groups form a site for the binding of Aβ (Kakio et al., 2001; Fantini et al., 2013). Certain studies suggest that Aβ oligomers may undergo their effects through binding to LR and thus, causing clustering of proteins including glutamate receptors and cellular prion protein. The formation of these clusters may be vital for the toxic signaling mechanisms that lie behind synaptic dysfunction in AD (Rushworth and Hooper, 2011). Meanwhile, Tau protein is a microtubule-associated protein in the brain and a key regulator of neurons. Abnormally hyperphosphorylated tau protein is the hallmark of the formation of neurofibrillary tangles (NFTs; Nanjundaiah et al., 2021). Tau pathology is, as well, affected by lipid metabolism (Sato and Morishita, 2015). Normal tau proteins allow the assembly and the stabilization of the microtubules, whereas hyperphosphorylated tau disrupts microtubules forming NFTs (Iqbal et al., 2009). A study conducted on mice that expressed a mutant human tau, showed that the level of the cellular cholesterol was higher in neurons that were affected by tau pathology (Glöckner and Ohm, 2014). Cholesterol metabolism, once impaired, is involved in tau hyperphosphorylation (Maccioni et al., 2010).

As a conclusion, elevated midlife serum cholesterol level has an increased risk for developing AD later in life. These cholesterol levels in the circulation are believed to modify the levels of 27-OHC and 24SOHC, along with other related increases in cytotoxic agents like 25-OHC which aided the intracellular transport of Aβ peptides. Finally, lipids rafts have shown vital roles in contributing to the pathogenesis of AD.

### 3.5 Alteration of protein function by methylation and deregulation of gene expression by DNA methylation

Senile plaque, polymorphous beta-amyloid protein deposits, is one of the two AD lesions, referred to as amyloid β (Aβ) plaques. This is a product of Aβ-precursor protein (APP) proteolytic processing by many enzymes (Walker, 2020). If APP was cleaved by both α-secretase and γ-secretase, Aβ40 will be the product. However, if APP was cleaved by β amyloid cleaving enzyme (BACE) also named β-secretase and γ-secretase, Aβ42 will be formed. The work of Vassar et al. demonstrated a two-fold elevation in the activity and the level of BACE1 in AD patients (Vassar et al., 2009). The 42 amino acids long Aβ42 protein, has a high tendency to assemble and form an amyloid (extracellular deposits of fibrils). Coppedè discusses that Aβ peptide deposits are one of the crucial early discoveries in the etiology of AD (Coppede, 2010). A balance between the secretase activities is crucial in preventing deposits and senile plaque formation. Any mutation that may interfere with this balance will result in a high Aβ42 protein concentration, or Aβ42/Aβ40 ratio increase, without affecting Aβ42 production. Interestingly, Zhang et al. found that nonsteroidal anti-inflammatory drugs (NSAIDs) seem to reduce the risk of AD (Zhang et al., 2018). The mechanism behind this is still not clear, but it seems that NSAIDs repress BACE1 promoter activity; hence decreasing β-secretase enzymatic activity with a decrease in senile plaque deposition (Cole and Vassar, 2008).

### 3.6 DNA methylation and AD

Scientists noticed that even patients with similar genetic backgrounds presented different pathologies in AD (Yokoyama et al., 2017). At the same time, epigenetic studies conducted found a correlation between epigenetic changes and AD (Pi et al., 2020). This pushed scientists to find the existing correlation. Pi et al. defined epigenetics as the study of the alteration in gene expression due to genetic modifications (Pi et al., 2020). Shafik et al. enhance the idea of how epigenetics add a layer of control in gene regulation (Shafik et al., 2022). Epigenetics is not only confined to DNA methylation but also includes RNA methylation, histone modification/modeling, and modifications of DNA-binding proteins (Barros and Offenbacher, 2009). We will be focusing here on the role of DNA methylation, gene expression, and protein methylation.

DNA methylation is considered the best representation of epigenetic modification since it maintains the cell’s function and affects its genetic expression. In AD, DNA methylation was found to support cellular processes, and synaptic plasticity in the CNS (Nikolac Perkovic et al., 2021). The authors also identified the idea that cytosine-phosphate-guanine motif (CpG islands) which are found mainly in regulatory regions are the target of most DNA methylation and gene expression. Yokoyama et al. cited how this methylation procedure is catalyzed by DNA methyltransferase (DNMT) enzymes which transfer a methyl group from SAM to the DNA (Yokoyama et al., 2017). As a result, we will have S-adenosylhomocysteine (SAH) from SAM; the major methyl donors (Pi et al., 2020). Once DNA methylation silences the target gene expression by preventing the binding of the transcription machinery (Dhar et al., 2021). To this day, the reported data regarding DNA methylation and its correlation to AD are inconsistent and inconclusive (Nikolac Perkovic et al., 2021). We will present a few examples of brain regions that have shown epigenetic modification in Alzheimer’s.

The frontal cortex is the primary cognition center and governs various abilities, including motor performance, task execution, thinking, and creativity (El-Baba and Schury, 2023). As a result, pathologies affecting this cortex contribute to the cognitive decline observed in patients with AD (Kalecký et al., 2022). Yokoyama et al. demonstrated the severe impact of AD on this cortex through a significant loss of synapses in affected patients (Yokoyama et al., 2017). Additionally, Mastroeni et al. attributed the cognitive decline in AD to a decrease or impairment in DNA methylation within the frontal cortex. However, Coppieters et al. and Rao et al. presented contrasting findings, revealing increased DNA methylation in AD patients. Specifically, Coppieters et al. identified higher methylation levels in the middle gyrus of AD patients compared to cognitively normal controls.

Within the Temporal cortex, Mastroeni et al. found that there was a significant decrease in DNA methylation in AD compared to the control group. Contrarily, Coppieters et al. showed that individuals with AD have higher levels of DNA methylation than cognitively normal controls. Furthermore, Lashley et al. found no significant difference in DNA methylation in the entorhinal cortex of patients with AD compared to control (Lashley et al., 2015).

The Hippocampus, a complex brain structure, has multiple functions such as learning, memory encoding and consolidation, and spatial navigation (Anand and Dhikav, 2012). Yokoyama et al. stated that hippocampus and cerebral atrophy represent one of AD hallmarks (Yokoyama et al., 2017). Chouliaras et al. found that AD patients have a decrease in DNA methylation in the hippocampus compared to a control (Chouliaras et al., 2013). In contrast, Bradley-Whitman et al. mentioned that higher levels of DNA methylation were observed within the hippocampus in AD patients (Bradley-Whitman and Lovell, 2013).

DNA methylation in Alzheimer’s has been shown to target specific genes (Pi et al., 2020). These specific genes are those associated with AD pathology such as APP, PSEN-1, microtubule-associated protein tau (MAPT), glycogen synthase kinase 3 beta (GS3K-β), protein phosphatase (PP2A), and apolipoprotein E (APOE; Yokoyama et al., 2017). Barrachina et al. found that there are no significant differences in DNA methylation of these genes between AD and control (Barrachina and Ferrer, 2009). On the other hand, Foraker et al. mentioned that having the ɛ4 in APOE will change the DNA methylation of the APOE gene, thus, contribute to AD susceptibility (Foraker et al., 2015). Yokoyama et al. added that APOE- ɛ4 is a risk factor, and that patients that are homozygous to this allele will be 8 times more prone to develop AD (Yokoyama et al., 2017). In addition, Kalecký et al. mentioned that the majority of APOE- ɛ4 carriers have AD (Kalecký et al., 2022). However, Karlsson et al. discuss that hypermethylation takes place in the promoter region of the APOE gene, and not the gene itself, which will result in an increased risk of dementia and AD (Karlsson et al., 2018). Sonawane and Chinnathambi showed that upregulating the GS3K-β promoter by demethylation and downregulating the PP2A promoter by methylation accelerated Tau hyperphosphorylation, thus increasing the risk of AD (Sonawane and Chinnathambi, 2018). Both PP2A and GS3K-β are regulatory enzymes of tau phosphorylation. PP2A is the primary Tau phosphatase, thus, when downregulated, a decrease in phosphatase activity is seen, which will increase the chance of Tau hyperphosphorylation (Sontag and Sontag, 2014). In addition, over activation of the kinase GS3K-β increases the chance of tau hyperphosphorylation and NFT formation. Moreover, over-activity of GS3K-β lead to memory impairment and increased senile plaque formation (Hooper et al., 2008). It is highly probable that epigenetic modifications such as methylation and demethylation of CpG are associated with amyloid plaques and NFT deposition which facilitate the development of AD and its progression.

## 4 1C metabolism and Parkinson’s disease

With the increasing life span of humans over the past few decades and the consequent aging population, neurological disorders have emerged as a primary cause of morbidity and mortality worldwide (Feigin et al., 2017). Parkinson’s disease (PD) is the most prevalent movement disorder and the second most prevalent chronic neurodegenerative disorder. Although the disease is rare prior to the age of 50, it affects 1% of the elderly above 60 years and detriments a total of 1–2% of the population at any given time (de Rijk et al., 2000; Hirtz et al., 2007). Both Parkinson’s disease’s late onset of symptoms and diagnosis, combined with an increasingly aging population, result in a predicted growth of the number of individuals affected by PD (Bach et al., 2011; Department of Economic and Social Affairs Population Division et al., 2013). This multifactorial geriatric neurodegenerative disorder presents with motor symptoms including tremors, posture instability, rigidity, and bradykinesia. PD may include as well as mental (non-motor) symptoms including depression and intellectual dysfunction (Kumudini et al., 2014; Miranda-Morales et al., 2017). Although the cause of the disease is not yet fully understood, there are two main hallmarks of PD. These hallmarks are the presence of ubiquitin-positive inclusion bodies containing α-synuclein protein also known as Lewis bodies and the excitotoxicity of dopaminergic (DA) neurons within the substantia nigra of the midbrain and the consequent loss of striatal dopamine. Both genetic and environmental factors contribute to the development of these hallmarks could potentially cause PD and the characteristic range of motor impairment (Forno and Singleton, 1996; Dunnett and Björklund, 1999; Murray and Jadavji, 2019).

### 4.1 Polymorphisms in 1C metabolism and Parkinson’s disease development

In recent years, extensive observational and genetic screening studies have sought to identify mutations and specific risk loci associated with PD. Several of these studies have explored the combined impact of epigenetic dysregulation and environmental factors, along with the genetic component, on the onset and advancement of PD (Cheon et al., 2012; Coppede, 2012). Some approaches currently used in research to study PD include post-mortem and human studies (Iacono et al., 2015). Polymorphisms in OCM genes and enzymes have been shown to increase the risk of PD development (Murray and Jadavji, 2019). Although the effect of these genes varies among different populations and ethnic groups, a common feature of these polymorphic genes is their ability to induce increasing homocysteine levels, which has been associated with the development and progression of PD (Rodriguez-Oroz et al., 2009; Levin et al., 2010; Saadat et al., 2018; Dong and Wu, 2020). Hcy, a sulfur-containing amino acid, is an important intermediate in the activated methionine cycle, which is directly intertwined with the folate cycle to constitute one-carbon metabolism, and thus any alterations in the levels of the metabolic intermediates of one of these cycles can affect Hcy levels (Ducker and Rabinowitz, 2017). Furthermore, polymorphisms in OCM enzymes may affect Hcy accumulation. High amounts of Hcy are a major risk factor for atherosclerosis and vascular disease, making PD patients at high risk of these conditions (Kuhn et al., 1998; Castro et al., 2006). In contrast, other studies have displayed an absence of correlation between Hcy and PD development (Li et al., 2020; Zhao et al., 2021). The aforementioned conflicting results thus require further investigation to clarify the type of relationship between those two factors.

Among the most studied polymorphisms of OCM enzymes associated with PD is the C677T variant of the gene encoding the enzyme MTHFR (methylenetetrahydrofolate reductase; de Lau et al., 2005; Gorgone et al., 2012; Wu X. et al., 2013). In an Italian case–control study performed on a sample size of 142 individuals, MTHFR C677T was found to be notably more frequent in PD patients when compared to the healthy control group (Gorgone et al., 2012). Moreover, in a prospective study done in the Netherlands that followed 5,920 individuals, homozygous mutant variants of the C677T polymorphism were found to be correlated with an increased risk of PD in smokers (de Lau et al., 2005). A cohort study involving 1,024 individuals with the aim of investigating the role of the MTHFR gene in the occurrence of PD in the Chinese population revealed a protective role of the C677T variant against PD development. Analysis of the A-T haplotype of the A1298C variant (another common MTHFR variant) in the same study, also expressed the same protective role (Yuan et al., 2016). However, more recent studies did not find evidence supporting the correlation between the MTHFR C677T polymorphism and PD. One case–control polish study, with a sample size of 574 individuals, found no significant correlation between the two factors and revealed no distribution differences of the wild-type MTHFR allele between the PD and control groups (Białecka et al., 2012). Another case–control study out of Turkey, involving 95 individuals, found no allele distribution difference of the MTHFR C677T gene between PD patients and the control group (Ay et al., 2022).

Further investigating into the presence of the MTHFR C677T variant revealed substantial allelic frequency and effect variation differences between different ethnicities (Guéant-Rodriguez et al., 2006). This was a significant revelation, as different analytical studies classified for different ethnicities reveal varying results (Gorgone et al., 2012). Moreover, two meta-analyses substantiated increased susceptibility to PD associated with the T wild-type allele in the European population. Yet, the same two studies each showed contradicting results for positive and negative associations found in Asian populations (Wu Y. L. et al., 2013; Zhu et al., 2015). When stratifying by ethnicity, a significant correlation between the MTHFR C677T dominant model and PD in a Caucasian population was revealed. Nonetheless, no correlation was in the Asian subgroup. Furthermore, upon evaluating the link between the MTHFR A1298C variant and PD, an increased risk of PD was observed in association with both the dominant and heterozygous models (Liu et al., 2018). These incongruous results regarding the correlation between MTHFR variants and PD may be reconciled by the differences in genetic background, epigenetic modification events, and environmental factors (Miranda-Morales et al., 2017).

Another folate cycle enzyme that was found to be associated with PD is MTRR (Methionine Synthase Reductase). The A1049GG polymorphism of the gene encoding this enzyme was significantly correlated with PD in a case–control study that revealed a greater risk of PD in the presence of other OCM polymorphisms, including MTHFR C677T (Fong et al., 2011). Finally, the literature so far has provided conflicting results about the association between these 1-C metabolism genes and PD. The recent evidence of the influence of epidemiological differences on such associations warrants further investigation into these likely associations.

### 4.2 B vitamins and Parkinson’s disease development and onset

In relation to OCM, there is growing evidence of a possible role of B-vitamins in PD onset and development, especially in patients with cognitive impairment (Seidl et al., 2014; McCarter et al., 2019). This potential role is supported by the fact that folate, the natural form of vitamin B9, and the synthetic food additive folic acid are essential cofactors in homocysteine metabolism. In addition, deficiencies in the levels of folate are associated with HHcy in PD patients (dos Santos et al., 2009). In a similar manner, B6 and B9 vitamin deficiencies lead to elevated Hcy levels and thus potentially to PD (Saadat et al., 2018; Dong and Wu, 2020; Kaye et al., 2020).

The correlation between B-vitamins levels and the risk of PD has been studied in models of vitamin deficiency and/or supplementation. Extensive data has been generated on the relationship between lower B vitamin serum levels and the risk of PD. A Japanese case–control study, with 617 participants, found that PD patients appeared to have lower levels of B6 and B12 vitamins compared to the control group. However, when adjusted for confounding variables, these levels were not found to be correlated with an increased probability of PD development (Murakami et al., 2010). However, chronic late-stage PD patients revealed a correlation between lower levels of B12 vitamin and the development of motor impairments and intellectual dysfunction. A prospective cohort study in advanced PD patients implicated vitamin B12 deficiency in the motor impairments, whereas the associated high Hcy levels were responsible for the intellectual dysfunction that developed (Christine et al., 2018).

The effect of dietary B vitamin supplementation on decreased PD likelihood was inconsistent. A meta-analysis including 10 studies revealed a correlation between high pyridoxine (vitamin B6) intake and a lower chance of PD development (Shen, 2015). Similarly, a prospective cohort study, with 5,920 participants done in the Netherlands, reported an association between lower pyridoxine (vitamin B6) intake and an increased chance of PD development, specifically in smokers. The same study revealed no discernible effects of dietary B9 or B12 vitamin intake on the risk of PD (Lau et al., 2006). While yet another prospective cohort study from the United States concluded that lower Parkinson’s development risk is independent of higher B6, B9, and B12 vitamin dietary intake (Chen et al., 2004). These inconsistencies among the studies may be attributed to the genetic and metabolic variation among individuals, and the disproportionality of serum B vitamin levels and consumption. Moreover, human error and personal biases cannot be excluded when utilizing questionnaire-based approaches (Murray and Jadavji, 2019).

### 4.3 Reduction of methyl groups from folate metabolism and its impact on gene expression in PD

The incorporation of a methyl group into the DNA strand, most typically to the C5 carbon atom on a cytosine ring, is referred to as DNA methylation (Moore et al., 2013). Although this epigenetic modification retains the nucleotide sequence intact, it silences or activates transcription and gene expression. This process is necessary for embryonic development, genomic imprinting, X-chromosome inactivation, and chromosome stability maintenance (Razin and Cedar, 1991).

DNA methylation is catalyzed by DNA methyltransferases (DNMTs), an enzyme that acts along the promoter region of a gene, known as “CpG Islands.” Unmethylated CpG islands have been associated with transcriptional activity, whereas methylated CpG islands have been linked to transcriptional repression (Moore et al., 2013). Several environmental factors, including diet, impact the pattern of DNA methylation. Additionally, gene expression can be disrupted by aberrant DNA methylation (Mahmoud and Ali, 2019). Accumulation of epigenetic modifications, throughout a lifetime, may contribute to the pathogenesis of late onset neurodegenerative diseases such as Parkinson’s disease (Kaut et al., 2022).

Parkinson’s disease has a higher prevalence among males due to the methylation profile of sex-specific genes such as microtubule-associated protein tau (MAPT). Both Parkinson’s and Alzheimer’s disease have been linked to MAPT. The aberrant methylation of the MAPT gene may contribute to Parkinson’s disease by altering axonal cytoskeleton integrity. DNA methylation patterns exhibit sexual dichotomy. As compared to men, women’s leukocytes were shown to have higher levels of MAPT methylation in the gene’s promoter region (Pankratz et al., 2009). This hypermethylation lowers MAPT expression, which may explain, at least in part, why women have a reduced incidence of Parkinson’s disease compared to men (Coupland et al., 2014).

In a genome-wide investigation, DNA methylation patterns in the cortical tissue of 14 male Parkinson’s disease patients and 10 male healthy controls were examined. Furthermore, the methylation of candidate genes and cytochrome P450 2E1 (CYP2E1) in DNA from blood mononuclear cells from 259 Parkinson’s disease patients and 182 healthy controls, as well as skin fibroblasts from 10 Parkinson’s disease patients and 5 healthy controls, were compared. The methylation patterns in the brains of PD patients and healthy people differed, as did the methylation patterns of multiple putative genes involved in PD pathogenesis and oxidative stress. The demethylation of CYP2E1 found in the brain was not detectable in 10 of 12 peripheral blood or skin fibroblasts, indicating that CYP2E1 methylation is tissue specific. A relationship between SNCA (synuclein alpha coding gene) and CYP2E1 expression was also observed (Kaut et al., 2022). Increased CYP2E1 activity has been linked to the creation of harmful metabolites, which have been linked to the deterioration of dopaminergic neurons (DA; Feng et al., 2015). A separate study also discovered 20 genes that were differently methylated in PD patients’ blood samples compared to controls (Moore et al., 2014).

Parkinson’s disease epigenetic investigation has focused on SNCA gene methylation levels (Coppedè, 2021). α-Synuclein is a protein that plays a role in neurodegeneration in Parkinson’s disease patients. It is implicated in dopaminergic neurotransmission. This protein is involved in the regulation of dopamine production and homeostasis (Lotharius et al., 2002). Indeed, lower SNCA promoter and intron-1 methylation levels, as well as enhanced SNCA expression, have been seen in the substantia nigra and other brain regions of Parkinson’s disease patients (Coppedè, 2021). Treatment with a DNA methylation inhibitor reduced methylation of the SNCA region, and levels of α-synuclein mRNA and protein were elevated (Jowaed et al., 2010).

α-Synuclein and SAM levels were found to be inversely related in a study that looked at the potential clinical effects of reduced methylation in PD patients. Low SAM levels were associated with greater α-Synuclein levels. Greater methylation potential, as shown by greater SAM/SAH ratios, was also linked with better cognitive abilities (Obeid et al., 2009). Two potential explanations have been proposed for these observations. The first argument is that low SAM causes SNCA hypomethylation and a consequent rise in synuclein expression (Jowaed et al., 2010). The second hypothesis is that increased SAH may impede protein repair processes including α-Synuclein repair. This would result in increased accumulation and aggregation of damaged synuclein.

It is evident from the above discussion that the methylation status of a Parkinson’s disease patient differs from that of a normal patient. Moreover, OCM plays an important role in this methylation process and thus to the pathogenesis and progression of PD.

### 4.4 Mechanisms through which folate metabolism influence PD onset

The onset and development of several neurological illnesses, including PD, can be influenced by OCM. Primary cell cultures lacking folate exhibit neurodegeneration and increased ROS formation (Ho et al., 2003). Although decreasing Hcy production proved effective at reducing ROS levels, direct Hcy exposure resulted in the same effects (Duan et al., 2002). These results are consistent with the inverse connection between Hcy and folate on ROS levels. Furthermore, studies have shown that Hcy increased mitochondrial membrane depolarization in human dopaminergic cells when coupled with the pesticide’s rotenone or iron. This exposure resulted in elevated mitochondrial ROS levels, which were further aggravated by the presence of Hcy. Nevertheless, the levels of ROS decreased after treatment with an antioxidant or a DNA damage inhibitor.

The role of OCM in Parkinson’s was investigated in animal models. Folate-deficient diets in mice, combined with exposure to 1-methyl-4-phenyl-1,2,3,6-tetrahydropyridine (MPTP) led to decreased survival of dopaminergic neurons and a greater degree of motor impairment compared to mice maintained on a control diet (Duan et al., 2002). Direct infusion of Hcy into the substantia nigra pars compacta (SNc) had the same outcomes (Duan et al., 2002). When Hcy was administered alone, it did not yield the same outcomes as MPTP delivery. This observation implies that folate deficiency and elevated Hcy levels may not directly be cytotoxic, rather enhance the susceptibility to neurodegeneration. In contrast other studies have found Hcy to be directly detrimental to dopaminergic neurons (Lee et al., 2005; Imamura et al., 2007). Hcy increased apoptosis in rat’s primary mesencephalic cells, in a dose-related manner, in response to MPTP. Hcy’s negative effects were particularly pronounced in dopamine-containing cells compared to non-dopamine-containing cells. Similar findings were made by Lee et al. (2005), who found that Hcy decreased mouse locomotor activity and tyrosine hydroxylase immunoreactivity after chronic treatment over a 36-day period.

Hcy accumulation is affected by the administration of L-DOPA in mouse models of Parkinson’s (Bone and Taufek, 1980; Wagner et al., 1984; Daly et al., 1997). Experimentally, acute treatment of L-DOPA increased Hcy levels while lowering SAM levels. Additionally, mice that were fed a diet lacking in folic acid responded to L-DOPA more strongly than control animals. These findings highly suggest a potential interaction between the metabolism of folate and L-DOPA (Daly et al., 1997).

Patients on chronic L-DOPA therapy, both MPTP patients and non-MPTP, see significant increases in Hcy levels in the SNc (Bhattacharjee et al., 2016). According to Bhattacharjee et al. L-DOPA markedly increased Hcy levels in the SNc of mice treated with MPTP compared to animals not treated with MPTP (Bhattacharjee et al., 2016). The reduction in Tyrosine hydroxylase positive (TH+) cells was 51% in the MPTP plus L-DOPA group versus 47% in the MPTP alone group. The loss of DA cells in the SNc was therefore unaffected by the significant increase in Hcy. In contrast, Duan et al.’s findings showed an increase in MPTP-related neurotoxicity when Hcy levels were elevated through folate-deficient diets or directly injected (Duan et al., 2002). These discrepancies may be explained by variations in MPTP dosages between studies or the interval of time between MPTP administration and euthanasia in the preceding study (Duan et al., 2002; Bhattacharjee et al., 2016). It is possible that L-DOPA had additional effects on the decline of dopaminergic cells.

The impact of L-DOPA on methylation processes has also been investigated in experimental animal models. In one study, L-DOPA administration resulted in higher levels of SAH, lower levels of SAM, and lower SAM/SAH ratios (Liu et al., 2000). The brain is capable of synthesizing methyl groups, but when mice lack dietary folic acid in combination with L-DOPA, methionine levels in brain tissue and serum drop significantly (Ordonez and Wurtman, 1974). According to Miller et al. blocking catechol-o-methyltransferase (COMT) with the inhibitor Ro 41–0960 could prevent L-DOPA-induced changes in a rat model, including decreased SAM, and elevated SAH and homocysteine levels. Consuming methionine can also alleviate L-DOPA’s effects on methyl group depletion (Bone and Taufek, 1980). This supports earlier studies that suggested L-DOPA could affect gene expression. The impact of B-vitamin intake on PD pathology has also been investigated, despite the lack of strong evidence to support the claim that consumption of B vitamins lowers the prevalence of PD. In a rat model of PD, the effects of supplementation with different B-vitamin doses in various combinations were examined in terms of behavior and Hcy levels (Haghdoost-Yazdi et al., 2012). The results of the Rotarod rotational behavior tests revealed that rats given ten times the amount of folate typically found in a minimum essential diet performed better (Haghdoost-Yazdi et al., 2012). The groups who received five times the amount of a particular combination of B vitamins fared similarly to the control group on the rotarod. None of the experimental groups had reduced Hcy levels compared to control. Thus, the benefits of such supplementation extend beyond lowering Hcy levels (Haghdoost-Yazdi et al., 2012).

The impact of supplemental folate was studied in invertebrate models of early onset Parkinson’s. Srivastav et al. found that in homozygous drosophila, Parkin deficiency resulted in greater pupal mortality rate, a longer transition to later life stages, and decreased motor function (Srivastav et al., 2015). Interestingly, the Parkin gene has a novel recessive variant that causes decreased mRNA and no Parkin protein. Parkin is involved in the degradation of unfolded proteins and is linked to typical mitochondrial activity. In addition to these findings, the authors found a decreased level of ATP that induced elevated oxidative stress, diminished antioxidant activity and impaired mitochondrial functionality. Thus, flies that were subject to an effective dose of folic acid (10 and 250 μM) recovered at least partially. In these flies, lethality was decreased, more flies reached later life stages, and motor function was improved. Overall, these results suggest that supplementing with folate may help to minimize the effects of DA breakdown in PD.

## 5 Conclusion

In summary, this review focuses on the diverse role of one carbon (1C) metabolism in human health and illness. The interconnected network of pathways involved in 1C metabolism plays a role in supplying methyl groups for DNA methylation, synthesizing amino acids and maintaining redox balance. This metabolic process is closely linked to inflamation, aging and neurodegenerative disorders. The review emphasizes the significance of comprehending the equilibrium of 1C metabolism as its disruption can have effects on cellular function and contribute to the development of a multitude of diseases. Aging is associated with changes in 1C metabolism that may contribute to the decline in function and increased vulnerability to age related ailments. Additionally, disrupted 1C metabolism has been implicated in the onset and progression of conditions such, as AD and PD, where DNA methylation and homocysteine metabolism play pivitol roles. In conclusion, gaining an understanding of the intricacies of 1C metabolism has the potential to pave the way for possibilities in developing therapies and approaches to minimize the effects of these diseases on human well-being.

## Author contributions

AB: Writing – original draft, Writing – review & editing. YH: Writing – original draft, Writing – review & editing. RK: Writing – original draft, Writing – review & editing. YR: Writing – original draft, Writing – review & editing. SY: Writing – original draft, Writing – review & editing. LK: Writing – review & editing. HG: Writing – review & editing. AK: Writing – review & editing. SA: Project administration, Supervision, Writing – review & editing. FH: Conceptualization, Project administration, Supervision, Writing – review & editing.
